# Natural Deep Eutectic Solvents Combined with Supercritical Carbon Dioxide for the Extraction of Curcuminoids from Turmeric

**DOI:** 10.3390/ph17121596

**Published:** 2024-11-27

**Authors:** Anna Stasiłowicz-Krzemień, Julia Wójcik, Anna Gościniak, Marcin Szymański, Piotr Szulc, Krzysztof Górecki, Judyta Cielecka-Piontek

**Affiliations:** 1 Department of Pharmacognosy and Biomaterials, Poznan University of Medical Sciences, Rokietnicka 3, 60-806 Poznan, Polandagosciniak@ump.edu.pl (A.G.); 2 Center for Advanced Technologies, Adam Mickiewicz University in Poznań, Uniwersytetu Poznańskiego 10, 61-614 Poznan, Poland; marcin.szymanski@amu.edu.pl; 3 Department of Agronomy, Poznań University of Life Sciences, Dojazd 11, 60-632 Poznan, Poland; piotr.szulc@up.poznan.pl; 4 Department of Entomology and Environmental Protection, Poznan University of Life Sciences, Dąbrowskiego 159, 60-594 Poznan, Poland; krzysztof.gorecki@up.poznan.pl

**Keywords:** turmeric, curcumin, supercritical carbon dioxide, natural deep eutectic solvent, neuroprotection

## Abstract

Background: Curcuminoids, the bioactive compounds found in turmeric, exhibit potent antioxidant, anti-inflammatory, and neuroprotective properties. This study aims to enhance the extraction of curcuminoids from turmeric using environmentally friendly solvents supercritical CO_2_ (scCO_2_) combined with natural deep eutectic solvents (NADESs) in one process, and to evaluate the resulting biological activity. Methods: A Box–Behnken statistical design was applied to optimize scCO_2_ extraction conditions—pressure, CO_2_ volume, and temperature—to maximize curcuminoid yield. Next, the menthol and lactic acid NADESs were selected, and these two solvents were combined into a single turmeric extraction process. The biological activity of the resulting extract was evaluated using antioxidant assays (ferric reducing antioxidant power and 2,2-diphenyl-1-picrylhydrazyl) and enzyme inhibition assays (acetylcholinesterase, butyrylcholinesterase, and tyrosinase). Toxicity assessments were conducted on the aquatic invertebrates *Daphnia pulex*, *Artemia* sp., and *Chironomus aprilinus*. Results: The most effective extraction was achieved using a menthol–lactic acid NADES as a cosolvent, integrated at a 1:20 ratio of plant material to NADESs while in combination with scCO_2_. The optimized scCO_2_–NADES extraction resulted in a high curcuminoid yield (33.35 mg/g), outperforming scCO_2_ extraction (234.3 μg/g), NADESs ultrasound-assisted extraction (30.50 mg/g), and alcohol-based solvents (22.95–26.42 mg/g). In biological assays, the extract demonstrated significant antioxidant activity and effective inhibition of enzymes (acetylcholinesterase, butyrylcholinesterase, and tyrosinase). Toxicity studies showed a concentration-dependent response, with EC_50_ for *Chironomus aprilinus* at the level of 0.098 μL/mL and *Daphnia pulex* exhibiting high sensitivity to the extract. Conclusions: This study highlights the potential of combining NADESs and scCO_2_ extraction in one process, demonstrating the effectiveness of scCO_2_–NADES extraction in maximizing curcuminoid yield and enhancing bioactivity.

## 1. Introduction

Turmeric (*Curcuma longa* L.) belongs to the ginger family (*Zingiberaceae*). It is cultivated in subtropical and tropical climates, and both India and China play a large role in its cultivation. It is appreciated not only for its taste but, above all, for its wide range of biological activities. In the rhizome of turmeric, 235 compounds have been identified, the main part of which are polyphenols and terpenoids [1]. The most important in the group of polyphenols are curcuminoids, where almost 80% is curcumin [1]. The next curcuminoids are demethoxycurcumin, constituting ~17%, and bisdemethoxycurcumin, approx. 3–6% [2]. The plant owes its aroma to monoterpenes and sesquiterpenes, which include eucalyptol, borneol, sabinene, α-phellandrene, germacrone, turmerone, curcumenone [3]. In the essential oils from flowers and leaves, monoterpenes dominate, while from rhizomes and roots—sesquiterpenes [1]. Steroids such as stigmasterol, β-sitosterol, cholesterol, and anthraquinone can also be distinguished. The chemical composition of compounds in turmeric rhizomes is influenced by several factors, including the plant’s variety and cultivation conditions [4]. Elements, such as climate, soil quality, and geographical location, play a significant role in shaping the chemical profile of the active ingredients found in the plant.

Turmeric has a wide range of therapeutic effects, including antioxidant, anti-inflammatory, anticancer, neuroprotective, and antidiabetic properties [4]. Turmeric exerts neuroprotective effects by mitigating oxidative damage and inflammation in brain cells, potentially slowing the onset and progression of neurodegenerative diseases such as Alzheimer’s and Parkinson’s, and promoting cognitive resilience [5]. Turmeric is also effective in regulating glucose levels and improving pancreatic cell function, which suggests its potential in diabetes therapy [6]. In the context of oncology, curcumin affects the signaling pathways of cancer cells, inhibiting their proliferation and supporting apoptosis processes. The biological effects of curcumin also include modulation of the immune system and inhibition of enzymes associated with the inflammatory response [7]. Turmeric has been shown to support digestive health by reducing inflammation, promoting bile production, and alleviating symptoms of indigestion and irritable bowel syndrome (IBS) [8].

Many methods of extracting turmeric have been studied. Common extraction methods, such as Soxhlet extraction, solvent extraction, and maceration, are widely used due to their simplicity, but have notable drawbacks [9]. These techniques are time-consuming, require large amounts of organic solvents, and often lack selectivity. The use of organic solvents raises environmental and health concerns due to their toxicity, volatility, and disposal challenges. Green extraction methods have emerged, focusing on reducing or eliminating harmful solvents while improving efficiency.

For more advanced methods of extraction, we can include ultrasound-assisted extraction (UAE), microwave-assisted extraction (MAE), ionic liquid extraction, enzyme-assisted extraction (EAE), and supercritical fluid extraction. Advances in extraction techniques have significantly improved the recovery and yield of bioactive compounds like curcumin from natural sources. UAE uses ultrasonic waves to create cavitation, enhancing molecular movement and extraction efficiency, with optimized conditions yielding notable improvements over conventional solvent methods [10]. MAE, which leverages localized heating, provides a fast, efficient alternative [11], while ionic liquids as a friendly solvent present an option with high thermal stability and low volatility, enhancing yields in both UAE and MAE [12]. In recent years, these techniques have become vital in reducing environmental impact, as they reduce reliance on harsh solvents. EAE employs specific enzymes to break down cell walls, improving access to active compounds [13], and pressurized liquid extraction (PLE) uses high temperature and pressure to increase solubility and extraction rates [14]. As demand grows for sustainable and effective natural compound extraction, these methods continue to gain importance, offering scalable, eco-friendly options for various industrial applications. Together, these innovative techniques demonstrate a shift toward sustainable, efficient extraction processes, enabling enhanced bioactive compound recovery for therapeutic and industrial applications.

Another eco-friendly advanced method of extracting plant materials is supercritical carbon dioxide extraction (scCO_2_). This type of extraction is performed at high temperature and pressure, above the critical point, when the fluid becomes supercritical and combines the properties of a liquid and a gas [15]. The scCO_2_ primarily dissolves low-polarity molecules, such as terpenes and essential oils. For example, after the optimization of scCO_2_ turmeric extraction (425 bar, 55–75 °C), the recovery turmerone is similar to n-hexane and methanol, and is in the range of 0.66–0.86% [16]. However, since curcuminoids are polyphenolic compounds with higher polarity, the addition of a polar cosolvent can enhance the extraction efficiency of turmeric by increasing the solubility of more polar molecules alongside the terpenes. This approach improves the overall yield of curcuminoids from *Curcuma longa*. A more polar solvent might be used as another step of extraction, as proposed by Martinez-Correa et al. [17]. A two-step extraction process using scCO_2_ followed by ethanol or water extraction yielded the highest total yield (23.4%) when water was used in the second step, while ethanol provided higher curcumin concentrations and antioxidant activity. Water soaking of *Curcuma longa* rhizomes prior to extraction significantly enhanced curcumin yield [18]. Polar solvents might also be used directly during the scCO_2_ extraction process. The use of ethanol as a cosolvent in scCO_2_ improved the extraction yield to 10.4% and curcumin recovery to 3.2% [19].

An alternative for organic solvents can be deep eutectic solvents (DESs), which are a class of green solvents formed by mixing two or more components, a hydrogen bond donor, and an acceptor, which interact to create a low-melting mixture. Due to their tunable properties, low toxicity, and biodegradability, DESs offer a promising alternative to traditional organic solvents in extraction processes, providing environmentally friendly and efficient solutions for the isolation of bioactive compounds [20]. Natural deep eutectic solvents (NADESs) are composed of naturally derived substances, such as sugars, organic acids, amino acids, and other biocompatible compounds [21]. We distinguish hydrophilic and hydrophobic eutectic mixtures, which differ in the degree of binding to water. Most NADESs are characterized by good thermal and chemical stability, and they preserve the stability of extracted compounds [21]. Jeliński et al. examined the stability of curcumin in methanol and in a mixture of choline chloride and glycerol under artificial light [22]. After two hours, the concentration of curcumin in the methanol solution decreased to 5% of the initial concentration, while in NADESs, there was no degradation of the compound [22]. NADESs have already been used to extract many compounds from various plant materials, such as elderberry flowers, passion fruit peels, *Scutellaria baicalensis* stem bark, wild thyme herbal dust, among many others [23,24,25,26]. Among the plant materials studied for NADES extraction, turmeric has also been explored [27,28,29,30]. The literature also presents the implementation of NADESs in MAE of turmeric [31]. Thus, this study is based on the existing literature regarding NADES extraction, focusing on the preparation methods and molar ratios.

The aim of this work is to obtain an extract of turmeric with the highest possible content of curcuminoids and then examine its neuroprotective properties. The first phase of the investigation included the optimization of scCO_2_ extraction. A suitable eutectic mixture was selected as the extractant, and the extract with the highest amount of active compounds was determined. The second phase of the research was to combine extraction with scCO_2_ and the eutectic mixture. Then, tests were performed to confirm antioxidant activity (DPPH and FRAP) and enzymatic tests (ability to inhibit acetylcholinesterase (AChE), butyrylcholinesterase (BChE), and tyrosinase). Biotoxicity studies were also performed. To the best of the authors’ knowledge, no existing research has combined the use of NADESs and scCO_2_ in a single, simultaneous extraction process of plant material, specifically for turmeric, highlighting the novelty of this approach.

## 2. Results

### 2.1. Extraction of Curcuminoids

**Figure 1:**
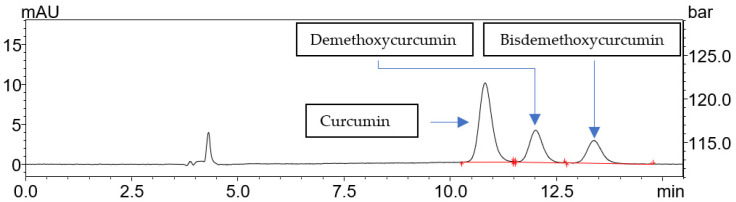
The chromatogram of curcuminoids present in turmeric extracts.

The Box–Behnken statistical design was used to optimize scCO_2_ extraction conditions for maximizing curcuminoid yield from turmeric. The content of curcuminoids was studied with the use of high-performance liquid chromatography (Figure 1).

The Design of Experiments (DoE) study focused on evaluating the effects of three critical factors: extraction pressure, CO_2_ volume, and temperature. Experiments were conducted across a range of conditions, with pressures set at 2500, 4500, and 6500 PSI (172.36, 310.26, 448.16 bar); CO_2_ volumes of 25, 100, and 175 mL; and temperatures at 30, 55, and 80 °C. Throughout this approach, 15 various scCO_2_ turmeric extracts were obtained, enabling a detailed analysis of how each variable impacts curcuminoid concentration (Table 1), ultimately identifying optimal parameters for the extraction process.

**Figure 2:**
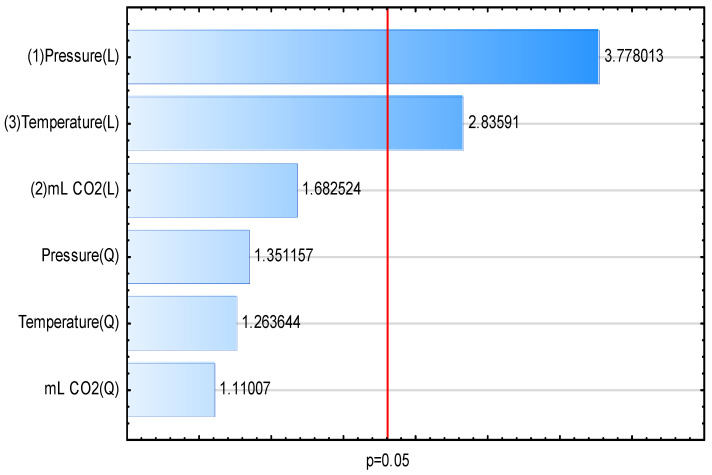
Pareto chart of standardized effects of Box–Behnken experimental analysis for curcuminoid content in the extracts.

**Figure 3:**
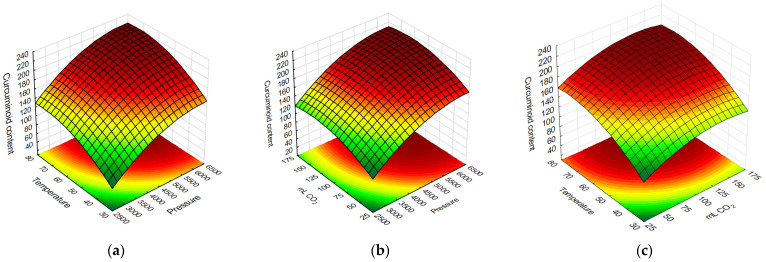
Response surface curve illustrating the effect of pressure to temperature (**a**), pressure to CO_2_ volume (**b**), and CO_2_ volume to temperature (**c**) on curcuminoid content.

A Pareto chart illustrating the curcuminoid content in the extracts displayed the standardized effects in descending order of their absolute values (Figure 2). The linear effects of pressure and temperature were significant at *p* > 0.05. However, the linear and quadratic effects of CO_2_ volume, as well as the quadratic effects of temperature and pressure, were not significant at *p* > 0.05. The response surface curves (Figure 3) visualize that higher values of temperature and pressure during extraction led to the highest curcuminoid content in the extracts. Based on these results, a temperature of 80 °C and a pressure of 6500 PSI (448.16 bar) were chosen as optimum extraction conditions. The amount of CO_2_ was found to be insignificant, so an intermediate value of 100 mL was used. Extraction under these conditions allowed us to obtain an extract with a content of 234.3 ± 5 μg/g.

In order to select the most effective cosolvent to be used in a further step, the raw material was subjected to ultrasound-assisted extraction for 30 min at a temperature of 30 ± 2 °C. The curcuminoid content obtained with the use of various extractants is presented in Table 2.

Among the NADESs used for the UAE, NADES_5 (menthol and lactic acid, 1:2 molar ratio) demonstrated the highest curcuminoid content in the extract, with 30.50 mg/g, making it the most effective in extracting these compounds. By comparison, standard alcohol solvents, 80% ethanol, and 80% methanol, resulted in curcuminoid contents of 26.42 mg/g and 22.95 mg/g, respectively. This indicates that NADES_5 outperformed the alcohol solvents in terms of curcuminoid extraction efficiency. Therefore, it was decided to combine scCO_2_ as a cosolvent with NADES_5 for optimal curcuminoid extraction.

**Figure 4:**
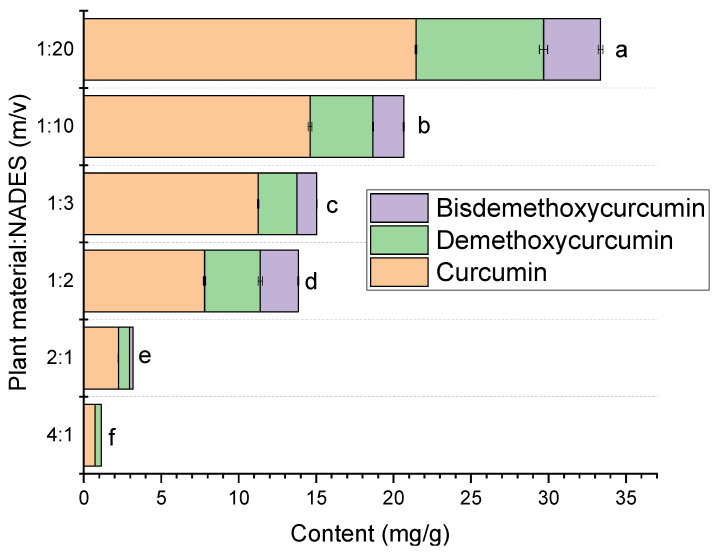
Curcuminoid content in the extracts was obtained using various ratios of plant material to eutectic solvent (*m*/*v*). Different letters (a–f) within the bars indicate statistical differences between curcuminoid content in the extracts (*p* < 0.05).

The next phase of the research involved integrating scCO_2_ extraction with NADESs. The curcuminoid content in the extracts increased with a higher proportion of eutectic solvent relative to the plant material (Figure 4). At the lowest ratio (4:1), curcuminoid levels were minimal, with curcumin content at 0.74 mg/g and no detectable bisdemethoxycurcumin. By contrast, the highest ratio (1:20) yielded significantly elevated curcuminoid concentrations, with curcumin at 21.45 mg/g, demethoxycurcumin at 8.23 mg/g, and bisdemethoxycurcumin at 3.67 mg/g. Statistical differences (*p* < 0.05) between various curcuminoid content suggested that increasing the eutectic solvent volume enhanced curcuminoid extraction efficiency across all extracts.

Statistical analysis was also performed to compare the curcuminoid content obtained under optimal conditions during scCO_2_ extraction (234.3 μg/g), the NADES_5 UAE extraction (30.50 mg/g), and the combined scCO_2_ and NADES_5 extraction (33.35 mg/g). The curcuminoid content achieved in the combined extraction (in CUR–scCO_2_–NADES_1:20), was significantly higher than in both extracts obtained in the earlier stages of the research.

### 2.2. Biological Activity Studies

**Figure 5:**
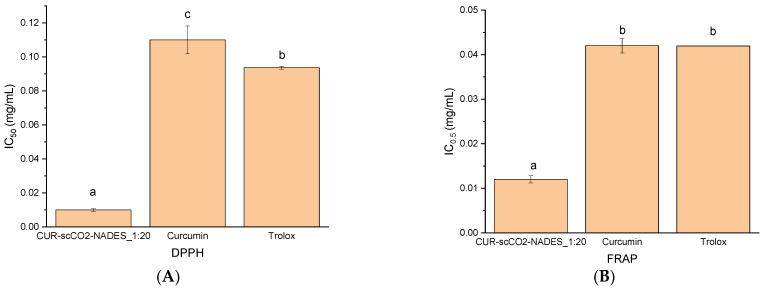
The antioxidant potential of the extract in DPPH (**A**) and FRAP (**B**) assays with references (curcumin, trolox). Different letters (a–c) within the bars indicate statistical differences (*p* < 0.05).

The antioxidant activity results, assessed by FRAP and DPPH assays, highlight the potency of CUR–scCO_2_–NADES_1:20. These assays are widely used to evaluate the ability of compounds to act as antioxidants by either reducing oxidants or scavenging free radicals. In both assays, CUR–scCO_2_–NADES_1:20 demonstrated superior antioxidant activity compared to references such as curcumin and Trolox, as it showed the lowest IC_50_ values (Figure 5). In the case of the FRAP assay, the IC_50_ value corresponds to the concentration required to reduce the ion Fe^3+^ (iron III) to Fe^2+^ (iron II) by 50%, and in the DPPH to scavenge free radicals by 50%. Lower IC_50_ values indicate stronger antioxidant activity, as a smaller concentration is needed to achieve the same effect.

**Figure 6:**
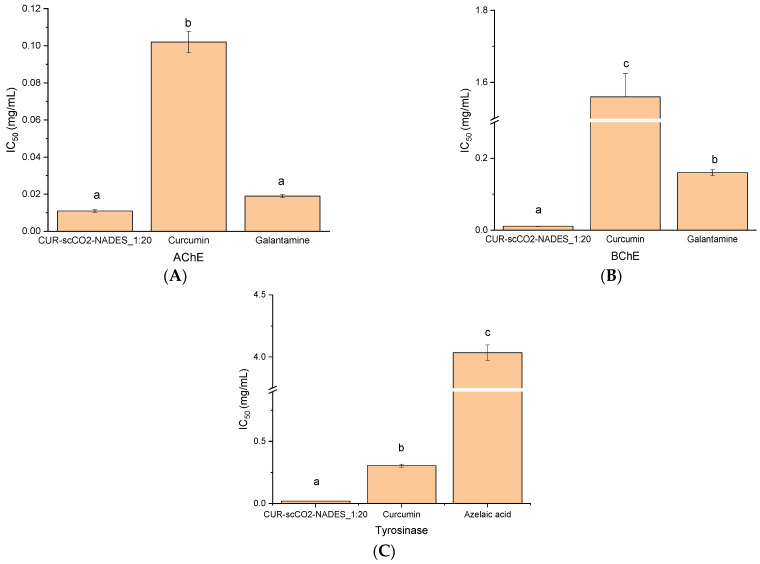
The potential of the extract to inhibit acetylcholinesterase (**A**), butyrylcholinesterase (**B**), and tyrosinase (**C**) with references (curcumin, galantamine, azelaic acid). Different letters (a–c) within the bars indicate statistical differences (*p* < 0.05).

The enzyme inhibition study shows that CUR–scCO_2_–NADES_1:20 effectively inhibits AChE, BChE, and tyrosinase, as IC_50_ values, are 0.01 mg/mL, 0.01 mg/mL, and 0.02 mg/mL, respectively (Figure 6). In AChE, it is statistically similar to galantamine activity, but in BChE and tyrosinase assays, it is significantly stronger than both references.

### 2.3. Biotoxicity Studies

**Figure 7:**
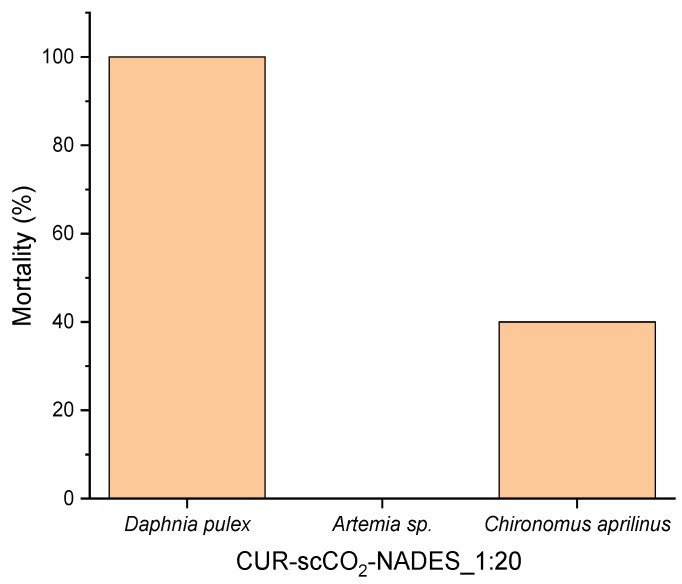
Percentage mortality of test organisms after 24 h of incubation with CUR–scCO_2_–NADES_1:20 (10 µL/mL).

The results of the preliminary toxicity study allowed the selection of two species, *Daphnia pulex*, and *Chironomus aprilinus*, for detailed studies (with a wide range of concentrations—0.04, 0.10, 0.20, 1.00, 4.00, and 10.00 µL/mL) (Figure 7). Due to the lack of a toxic effect of the extract on *Artemia* sp., the use of these invertebrates in the study was abandoned.

**Figure 8:**
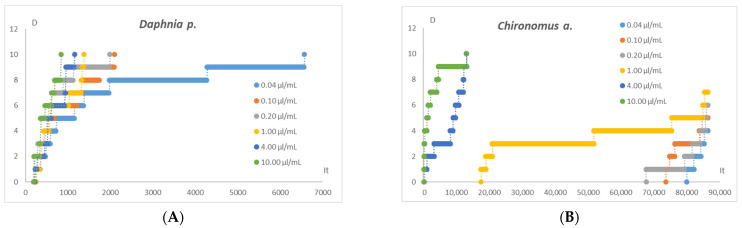
Graphs of the dependence of the number of dead organisms of *Daphnia p.* (**A**) and *Chironomus a.* (**B**) on the incubation time.

Biotoxicity main studies of the extract conducted on *Daphnia pulex* and *Chironomus aprilinus* showed that the samples were toxic to the test organisms to varying degrees. Table 3 presents the equations of logarithmic curves, correlation coefficients, and IT_50_ (s) coefficient values (time after which 50% death of test organisms occurred) for individual extract concentrations, and Figure 8 presents graphs of the dependence of the number of dead organisms on the incubation time.

Table 4 presents the determined EC_50_ coefficients (the concentration at which 50% of the test organisms died after 24 h of incubation), the logarithmic curve equation, and the regression coefficient R^2^.

The invertebrate more sensitive to the toxic effects of the extract was *Daphnia pulex*, as evidenced by the significantly shorter times in which the half-life of the organisms occurred and the lower concentrations of the extract causing the death of the organisms.

## 3. Discussion

*Curcuma longa* contains curcuminoids, including curcumin, demethoxycurcumin, and bisdemethoxycurcumin, which are responsible for most of its therapeutic properties, including neuroprotective effects. Changes occurring in the nervous system are often irreversible, and the disease itself is incurable, making it essential to search for preventive solutions. This research aimed to obtain an extract from *Curcuma longa* with the highest possible curcuminoid content and then investigate its neuroprotective properties.

Firstly, optimization of turmeric extraction with scCO_2_ was conducted with the use of DoE. The maximum yield of the three curcuminoids combined reached 0.32 mg/g under pressure parameters of 6500 PSI (448.16 bar) and a temperature of 80 °C. These parameters were found to have a significant impact on the curcuminoid content, whilst the CO_2_ volume was insignificant

Research in the literature presented obtaining the maximum curcuminoid content with scCO_2_ at the level of 0.45 mg/g under conditions of 350 bar and 65 °C; while in another study, the CO_2_ extract showed intermediate curcuminoid concentrations: 0.11% for curcumin, 0.02% for demethoxycurcumin, and 0.004% for bisdemethoxycurcumin [16,32].

To enhance the efficiency of the extraction process, eutectic mixtures were selected as potential cosolvents with the use of an ultrasound-assisted extraction process. DESs can be either hydrophilic or hydrophobic, depending on the nature of their constituent compounds, which influences their miscibility with water and other polar solvents [33]. Hydrophilic DESs typically contain hydrogen bond donors and acceptors with polar groups, while hydrophobic DESs are designed with nonpolar or weakly polar components to minimize water interaction. During this research, both types of DESs were proposed. To yield an extract with the highest active compound content among previously published DESs for extraction were prepared [31,34,35,36,37,38,39]. Eight NADESs were prepared with choline chloride and menthol as hydrogen bond acceptors, while urea, lactic acid, citric acid, urea, propylene glycol lauric acid, stearic acid, and myristic acid were used as hydrogen bond donors. The greatest results were obtained when turmeric rhizome was extracted using a menthol and lactic acid mixture (1:2) with an ultrasonic bath for 30 min at a temperature of 30 ± 2 °C, resulting in a curcuminoid content of 29.95 mg/g. Thus, it was further used in the combination of scCO_2_ as a cosolvent with a NADES. At this step, 80% of ethanol and methanol were also used to compare NADESs with traditional solvents. The extraction of curcuminoids from turmeric with a menthol and lactic acid mixture was more efficient than the one with alcohol. In a study by Oliveira et al. (2021), the same eutectic mixture was used to extract turmeric, although quantitative curcuminoid content was not characterized; instead, antioxidant activity was assessed using the DPPH model, reaching 41.0 mg Trolox/g extract, while the extract obtained in this research demonstrated activity of 10.35 µg Trolox/g extract [37]. In a study by Patil et al. (2021), the curcuminoid content in an extract prepared using a choline chloride and lactic acid mixture (1:1) reached up to 58.87 mg/g, and with a choline chloride and citric acid mixture (2:1), it was 54.43 mg/g [35]. By using a choline chloride and lactic acid mixture (1:1) with 20% water, researchers achieved 77.13 mg/g of curcuminoids in the extract [35]. The curcuminoid content in the eutectic mixture extract was higher than in ethanol and methanol extracts, which contained 26.34 mg/g and 22.93 mg/g, respectively. The differences in extraction efficiency can be attributed to the time of extraction, temperature, plant material of various origins, and different compositions of eutectic mixtures, whose polarity and pH influence the extraction of secondary plant metabolites and affect the stability and solubility of certain compounds. The biological activity of the extract will depend on the profile of compounds present, as different metabolites contribute distinct pharmacological properties.

Next, the scCO_2_ extraction was coupled with NADESs under the conditions established in the DoE. The ratio between plant material and eutectic solvent was studied between 4:1 to 1:20. A content of 33.35 mg/g was obtained with a raw material-to-eutectic mixture ratio of 1:20, which was the highest result among all studies conducted, outperforming significantly those achieved using an ultrasonic bath. The literature contains relatively few studies exploring NADESs and scCO_2_ as complementary green solvents. One of the literature references discusses the benefits of combining these solvents, as it enhances molecular mobility, modifies polarity, and preserves microstructure, thereby increasing the potential applications of DESs in chemistry and engineering [40]. This approach is also proposed in supercritical fluid chromatography, where NADESs are explored as green additives to replace conventional, often toxic, additives, thereby advancing both sustainability and efficiency in separation techniques [41]. The simultaneous combination of scCO_2_ as a cosolvent with NADESs during the extraction process is an innovative approach, as this combination, to the best of the authors’ knowledge, has not yet been used in the literature, especially for turmeric extraction. Vladić et al. used NADESs to disperse scCO_2_ extract of *Satureja montana* to stabilize the aroma of volatile organic compounds, such as carvacrol, thymol, and thymoquinone, with less degradation and oxidation compared to the control [42]. A sequential extraction approach was developed for *Lavandula stoechas*, using scCO_2_ followed by NADES ultrasound-assisted extraction, allowing for the targeted extraction of terpene and polyphenol fractions, with NADESs—especially betaine-ethylene glycol—enhancing the yield of bioactive compounds, thus offering both superior antioxidant and antimicrobial activities compared to conventional solvents [43]. NADESs were implemented in PLE alongside ultrasound-assisted extraction to enhance the extraction of phenolic compounds from Hass avocado residues [44]. The combination of NADESs with these extraction techniques increased the extraction efficiency, leading to high yields of phenolic compounds. DESs were also implemented in the microwave-assisted extraction of turmeric, with a choline chloride–citric acid mixture containing 30% water as the solvent, yielding 89.87 mg/g of curcuminoids [31]. Deep eutectic solvents (DESs) were utilized in the microwave-assisted extraction of turmeric, with a choline chloride-citric acid mixture containing 30% water as the solvent, yielding 89.87 mg/g of curcuminoids. Another study assessed the efficiency of subcritical water extraction and pressurized NADESs at varying temperatures for extracting pectin from passion fruit rinds and their residual biomass, with pressurized NADESs achieving the highest yields and pectin with superior structural characteristics compared to conventional extraction [45]. Sequential use of NADESs followed by scCO_2_ have also been applied in the extraction of essential oils from plant materials, where NADESs efficiently extracted the desired compounds, and scCO_2_ further enhanced the yield by stripping residual oils from the NADES phase [46].

In the next stage, biological activity studies of the CUR–scCO_2_–NADES_1:20 extract were performed. Antioxidant activity was determined using the DPPH and FRAP methods. In the DPPH study, IC_50_ = 0.01 mg/mL was obtained for the extract, which outran the trolox and curcumin (IC_50_ values were 0.09 and 0.11, respectively). The CUR–scCO_2_–NADES_1:20 extract also proved to be better than the referents in the FRAP method. (IC_0_._5_ 0.01 mg/mL). In the literature, for the aqueous extract of turmeric, researchers obtained the DPPH assay IC_50_ = 5.99 mg/mL, and for the ethanolic extract, IC_50_ = 0.12 mg/mL [47,48]. For the FRAP assay, the IC_0_._5_ of the aqueous extract of *Curcuma longa* was 9.8 mg/mL, and for the methanol extract, IC_0_._5_ = 23.8 mg/mL [49]. The potential of CUR–scCO_2_–NADES_1:20 extract to inhibit the enzymes connected to neurodegeneration was also studied, and the superiority of the extract was revealed in comparison to the references. In a study based on the ability to inhibit AChE, the IC_50_ value for the CUR–scCO_2_–NADES_1:20 was 0.01 mg/mL, whilst in BChE, the value was 0,01 mg/mL. According to the literature, curcuminoids inhibited AChE with an IC_50_ of 19.67 µM, while curcumin alone showed an IC50 of 67.69 µM [50]. Sudeep HV et al. also investigated the activity of an extract of *Curcuma longa*, which showed an IC_50_ of 139.2 µg/mL for AChE and 180.9 µg/mL for BChE [51]. Kalaycıoğlu et al. examined the activity of individual curcuminoids, with bisdemethoxycurcumin showing the best result with an IC_50_ of 2.14 µmol/L, followed by demethoxycurcumin with an IC_50_ of 19.7 µmol/L, and curcumin with an IC_50_ of 51.8 µmol/L [52]. In the case of tyrosinase inhibition, CUR–scCO_2_–NADES_1:20 was also stronger than the proposed references, curcumin, and azelaic acid, with IC_50_ of 0.02 mg/mL. Firmansyah et al. demonstrated this ability for an ethanolic extract of turmeric with an IC_50_ of 564.8 µg/mL [53]. The curcuminoid content in the raw material depends on its cultivation location and can range between 30 mg/g and 150 mg/g [54]. Due to this fact, it is difficult to directly compare the results obtained in current research with data in the literature because the authors of the scientific papers most likely worked with different raw materials with various curcuminoid content. When comparing the methods used by other researchers to those employed in this study, the best solution turned out to be a combination of scCO_2_ extraction and a eutectic mixture.

Biological assays in this study included various types of samples: blanks, controls, and control blanks, all conducted in vitro to exclude the impact of the solvent on the activity. Biological activity studies involving NADES-based extracts are typically conducted as part of an extract’s characterization, also containing menthol and lactic acid, using in vitro assays to assess their bioactivity and safety, as is the case with other types of extracts like hexane, acetone, or organic solvent-based extracts [37,55,56,57,58]. Performing in vitro activity studies on NADES-containing extracts aligns with standard practice, as demonstrated in studies where these evaluations have provided insights into the bioactivity and safety profiles of NADES-based extracts.

The NADES–scCO_2_ extract obtained in this study did not show biotoxicity against *Artemia* sp. at a concentration of 10 µL/mL; however, it exhibited some toxicity toward *Daphnia pulex* and *Chironomus aprilinus*, which warrants further investigation. More toxicity studies of the extracts obtained within this study could be conducted; for example, extracts of *Curcuma longa* using menthol and lactic acid prepared by ultrasound-assisted extraction as shown by another research group [37]. That extract also showed high inhibition of AChE and BChE activity, along with significant iron-chelating and antibacterial activities; for that, no genotoxicity or cytotoxicity was observed, as studied on *Allium cepa* cells.

Another study demonstrated that NADESs, composed of choline chloride and 1,4-butanediol, exhibited significantly lower toxicity compared to conventional organic solvents like hexane, toluene, and dimethylformamide [59]. In general, NADES are considered to be safe solvents [60]. NADESs can be formulated with various components acting as hydrogen bond donors and acceptors; while some NADESs are regarded as safer, others may pose a higher risk when used in human applications. NADESs containing organic acids are more toxic than those based on sugars and polyols, but this does not mean that all organic acid-based NADESs are toxic [61]. Thus, further safety evaluations of the extract obtained within the current study are recommended. Alternatively, NADESs from the extract should be further recovered. Numerous technologies have been investigated to recover and purify NADESs. These recovery and purification techniques encompass approaches such as anti-solvent addition, recrystallization, various extraction methods (liquid–liquid and solid–liquid), short path distillation, supercritical fluid extraction, separations based on density differences, and membrane filtration [62].

## 4. Materials and Methods

### 4.1. Plant Material and Chemicals

The dried and milled turmeric rhizome was obtained from Planteon, Żelazków, Poland (Batch number 25921/139/412/1). Curcumin (purity > 99.5%), demethoxycurcumin, and bisdemethoxycurcumin (≥98% purity) were purchased from Sigma–Aldrich (Poznań, Poland). Acetic acid, isopropanol, acetonitrile, sodium chloride, and sodium acetate trihydrate were supplied by POCH (Gliwice, Poland). Choline chloride, menthol, lauric acid, stearic acid, myristic acid, propylene glycol, AChE from *Electrophorus electricus*, BChE from equine serum, acetylcholine iodide, butyrylcholine iodide, magnesium chloride hexahydrate, 2,2-diphenyl-1-picrylhydrazyl, iron(III) chloride hexahydrate, 2,4,6-tripyridyl-s-triazine, 5,5′-dithiobis-(2-nitrobenzoic acid), L-DOPA, tyrosinase, Trolox, 2,4,6-tris(2-pyridyl)-1,3,5-triazine, and azelaic acid were all sourced from Sigma–Aldrich (Poznań, Poland). Urea and citric acid monohydrate were provided by Chempur (Piekary Śląskie, Poland), while lactic acid was obtained from Biomus (Lublin, Poland). Ethanol (analytical grade) and methanol (analytical grade) were acquired from Sigma–Aldrich, and J.T. Baker, respectively. Dimethyl sulfoxide was supplied by Chempur, with Trizma^®^ Base and Trizma^®^ hydrochloride also from Sigma–Aldrich. Distilled water was prepared using a Direct-Q 3 UV water purification system from Merck Millipore (Darmstadt, Germany).

### 4.2. High-Performance Liquid Chromatography

The content of curcuminoids was determined using a validated (Table S1) high-performance liquid chromatography (HPLC) with a UV detector. The determination was carried out using a stationary phase: Agilent LiChrospher RP-C18-5 column (250 mm × 4 mm) [63]. The mobile phase consisted of a mixture of isopropanol, acetonitrile, distilled water, and acetic acid in the ratio of 3:1.5:5:0.5 (*v*/*v*/*v*/*v*). The flow rate was set to 0.5 mL/min, and the detection wavelength was set at 420 nm. The injection volume was 10 µL. The results were obtained and processed by LabSolutions LC software (version 1.86 SP2, Shimadzu Corp., Kyoto, Japan).

### 4.3. Extraction of Curcuminoids

Using a DoE approach, specifically the Box–Behnken design, an experimental framework was developed to evaluate the impact of process conditions on the efficiency of scCO_2_ extraction from turmeric (Table 5). The study focused on three main variables: pressure, CO_2_ volume, and extraction temperature, with the concentration of curcuminoids as the primary outcome measure, quantified via HPLC. The scCO_2_ extraction of powdered turmeric was conducted in an SFT-120XW (Supercritical Fluid Technologies Inc., distrib. shim-pol, Izabelin, Poland) extractor in dynamic mode, with pressure and temperature varied between 2500 and 6500 PSI (172.37–448.16 bar) and 30 and 80 °C, respectively. The experimental setup, summarized in a table of fifteen extraction trials, involved maintaining constants, such as a turmeric mass of 7.0 ± 0.01 g. Results were analyzed using Statistica 13.3 software for statistical validation and optimization.

To yield an extract with the highest curcuminoid content, various DESs for which preparation and usefulness in extracting curcuminoids were previously published were prepared [31,34,35,36,37,38,39]. To obtain hydrophilic and hydrophobic eutectic mixtures, the hydrogen bond donors and acceptors were mixed in ratios presented in Table 6 at 70 °C until a clear liquid was obtained. To obtain the extracts, 0.25 g of plant material was weighed, and 5.0 mL of a eutectic mixture was added. Extracts based on 80% ethanol and 80% methanol were also prepared, following the same procedure as for the eutectic mixtures [64,65]. The extracts were then subjected to ultrasound treatment (constant, uninterrupted sonication, frequency 37 kHz, ultrasonic peak max. 800 W) (Thermo Fisher Scientific, Waltham, MA, USA) for 30 min at a temperature of 30 ± 2 °C. In the next step, the samples were centrifuged, and the curcuminoid content was determined using HPLC.

The next phase of the research involved integrating scCO_2_ extraction with NADESs. Powdered turmeric and the eutectic mixture were added to the extraction vessel in varying volumetric ratios to the raw material mass (Table 7). The scCO_2_ extraction was performed under 6500 PSI (448.16 bar) and 80 °C based on the DoE. Even though the amount of scCO_2_ was insignificant during the DoE study, it was decided to retain the amount of CO_2_ used during the extraction to 100 mL as in the cell was not only plant material but also NADES_5.

### 4.4. Biological Activity Studies

Two assays, the 2,2-Diphenyl-1-picrylhydrazyl (DPPH) and ferric-reducing antioxidant power (FRAP) methods were used to evaluate the antioxidant activity of the extract with the highest curcuminoid content. As a reference, trolox and pure curcumin were used.

#### 4.4.1. Antioxidant Activity Studies

The DPPH assay was carried out in a 96-well microplate with spectrophotometric analysis [66]. For this test, a 0.2 mM methanol solution of DPPH was used. To initiate the reaction, 25.0 µL of the sample was mixed with 175.0 µL of DPPH solution, followed by incubation in a dark environment at room temperature for 30 min with continuous shaking. Absorbance readings were taken at 517 nm using a Multiskan GO plate reader (Thermo Fisher Scientific, Waltham, MA, USA). A blank sample, consisting of the DPPH solution with the solvent only, was also measured at 517 nm. The extract’s own absorbance was also measured. The percentage of DPPH radical inhibition by the extracts was calculated using the following formula:

(1) $$DPPH\;scavenging\;activity\;(\%)=\frac{A_{0}-A_{i}}{A_{0}}\times 100\%$$

In the formula, *A*_0_ is the control sample absorbance, whilst *A_i_* is the test sample absorbance. Each measurement was conducted in six replicates. The IC_50_ values, which determine the concentration of the extract/reference that inhibits the formation of DPPH by 50%, were determined by linear regression analysis.

In the FRAP assay, colorless Fe^3+^ ions were reduced to Fe^2+^, forming a blue complex with 2,4,6-tris(2-pyridyl)-1,3,5-triazine (TPTZ) [67]. This reaction was tracked by measuring absorbance at 593 nm using a Multiskan GO plate reader after incubating a mixture of 25.0 µL of the extract or Trolox or curcumin solution with 175.0 µL of FRAP reagent (composed of 25 mL acetate buffer, 2.5 mL TPTZ solution, and 2.5 mL FeCl_3_·6H_2_O solution) in the dark at 37 °C for 30 min. Control samples and extracts were analyzed, and each measurement was performed in six replicates. The extract’s own absorbance was also measured. The IC_0_._5_ value corresponds to the concentration indicating 0.5 absorbance.

#### 4.4.2. Anticholinergic Activity Studies

The extract was assessed for its potential to inhibit enzymes associated with neurodegenerative conditions, specifically AChE and BChE.

The inhibition of AChE and BChE was evaluated using a method developed by Ellman et al. [68]. This assay employs synthetic substrates (thiocholine esters), which release thiocholine upon enzymatic reaction with 5,5′-dithio-bis-(2-nitrobenzoic) acid (DTNB), leading to the formation of the 3-carboxy-4-nitrothiolate anion (TNB anion), visible as a color change. The reaction was conducted in a 96-well plate, where each well contained 60.0 µL of 0.05 M Tris-HCl buffer (pH 8.0), 5.0 µL of the extract, and 30.0 µL of AChE/BChE solution at a concentration of 0.2 U/mL. The mixture was incubated with shaking at room temperature for 5 min. Following incubation, 30.0 µL of a 1.5 mM solution of either acetylthiocholine iodide (ATCI) or butyrylthiocholine iodide (BTCI), and 125.0 µL of a 0.3 mM DTNB solution was added to each well, with a further 20-min incubation under the same conditions. Blank samples (reaction mixtures without the enzyme and additional Tris-HCl buffer), control samples (solvent added in place of extract), and control blanks (control sample mixtures without enzyme but with an additional Tris-HCl buffer) were prepared. Galantamine and curcumin were used as the reference.

Absorbance was measured at 405 nm, and the percentage of inhibition of AChE and BChE by the extracts was calculated as follows:

(2) $$\mathrm{AChE}/\mathrm{BChE}\;\mathrm{inhibition}\;(\%)=\frac{1-(A_{1}-A_{1b})}{\left(A_{0}-A_{0b}\right)}\times 100\%$$

where:

*A*_1_—the absorbance of the test sample

*A*_1*b*_—the absorbance of the blank of the test sample

*A*_0_—the absorbance of control

*A_b_*—the absorbance of the blank of control

IC_50_ value was calculated, indicating the concentration of the extract needed to inhibit 50% of AChE/BChE activity.

#### 4.4.3. Tyrosinase Inhibitory Activity Studies

The tyrosinase inhibition assay measures a reduction in color intensity, which indicates the suppression of enzyme activity [24]. This assay relies on an inhibitor that blocks L-DOPA from reaching the active site of tyrosinase, effectively stopping the reaction. In a 96-well plate, each well was prepared by adding 75.0 μL of 0.1 M phosphate buffer (pH 6.8), 25.0 μL of the extract, and 50.0 μL of enzyme solution (192 U/mL), followed by incubation at room temperature with shaking for 10 min. After this initial incubation, 50 μL of 2.0 mM L-DOPA solution was added, and the wells were incubated again for 20 min under the same conditions. A blank for the test sample (without the enzyme, with additional phosphate buffer to adjust the volume) was prepared, along with a control sample (replacing the test sample with solvent), and a blank for the control sample (without enzyme and with extra phosphate buffer). Azelaic acid and pure curcumin served as references. Absorbance measurements of the samples were then taken at 475 nm, allowing for the determination of tyrosinase inhibition by the extracts using the following formula:

(3) $$\mathrm{Tyrosinase}\;\mathrm{inhibition}\;(\%)=\frac{1-(A_{1}-A_{1b})}{\left(A_{0}-A_{0b}\right)}\times 100\%$$

where:

*A*_1_—the absorbance of the test sample

*A*_1*b*_—the absorbance of the blank of test sample

*A*_0_—the absorbance of control

*A*_0*b*_—the absorbance of the blank of control

The IC_50_ value was calculated, indicating the concentration of the extract needed to inhibit 50% of tyrosinase activity.

### 4.5. Biotoxicity Studies

For biotoxicity studies, selected live food for aquarium fish was used (Manufacturer: IT-Ichthyo Trophic Ltd., Stare Polichno, Polska): water flea (*Daphnia pulex*), medium-sized bloodworm (*Chironomus aprilinus*), brine shrimp (*Artemia* sp.).

**Figure 9:**
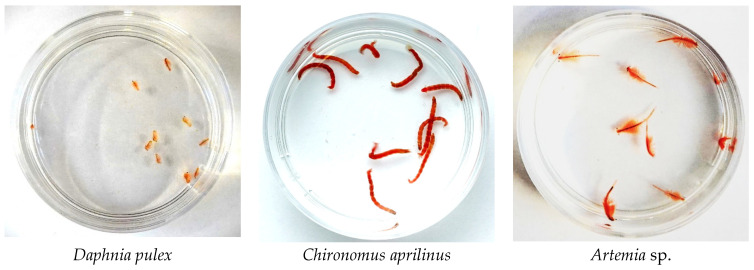
Petri dishes with test organisms—preliminary studies.

During preliminary biotoxicity studies, in six Petri dishes with a diameter of 9 cm containing 50 mL of aquarium water, the following number of test invertebrates were added: 50 organisms of *Daphnia pulex*, 30 organisms of *Chironomus aprilinus*, and 20 organisms of *Artemia* sp. Then, 0.5 mL of CUR–scCO_2_–NADES_1:20 (at a concentration of 0.054 mg/mL) was introduced, the mixture was homogenized, resulting in a concentration of 10.00 µL of eutectic mixture per mL of water in each dish (Figure 9). After 24 h, the number of live organisms was checked, and the survival rate of each tested organism was calculated. The study was conducted to determine the survival rate of invertebrate organisms after introducing a foreign substance, in this case, an extract, into their habitat.

In the main biotoxicity study, 4 cm diameter Petri dishes with ten invertebrates in 5 mL of aquarium water, 5; 20; 50 µL of the extract were added to obtain concentrations of 1.00, 4.00, and 10.00 µL/mL, and 9 cm diameter dishes with ten organisms in 50 mL of aquarium water, 2, 5, and 10 µL were added to obtain concentrations of 0.04, 0.10, and 0.20 µL/mL, respectively. Aquarium water was used for the study to avoid additional stress on the test organisms associated with a change in their living environment. The dishes were placed on a multimedia projector equipped with a camera (AVer SPB350+ No157, New Taipei, Taiwan). A 24-h image fixation cycle was set every 10 s. After the study was completed, the collected multimedia material was analyzed.

## 5. Conclusions

This study demonstrates the potential of combining natural deep eutectic solvents (NADESs) with supercritical carbon dioxide (scCO_2_) in a single extraction process for turmeric. By reducing reliance on organic solvents, this method supports sustainability goals and addresses the growing demand for eco-friendly extraction technologies. The integration of NADESs with scCO_2_ has proven to be a highly effective approach, significantly enhancing the yield of curcuminoids. Specifically, the use of menthol–lactic acid NADESs during scCO_2_ extraction showed superior performance, improving extraction efficiency compared to scCO_2_ alone, ultrasound-assisted NADES extraction, and alcohol-based solvent extraction. This combined “green” approach not only improved overall extraction efficiency but increased biological activity, particularly in terms of neuroprotective effects. Despite the promising results, several key limitations and future directions should be considered. While the study demonstrates the efficacy of this combined extraction method on a small scale, further research is needed to assess its scalability for industrial applications. This includes evaluating the feasibility of maintaining high extraction efficiency and cost-effectiveness at larger scales. Additionally, further safety and toxicity evaluations of the extract obtained in this study are necessary. Moreover, the recovery and purification of NADESs, which are integral to the extraction process, require further investigation. Exploring the effectiveness and cost-efficiency of these recovery methods will be crucial for optimizing the sustainability and economic viability of the process. By addressing these challenges and exploring these future directions, the scCO_2_–NADES extraction technique can be refined for industrial-scale applications, enhancing its potential for extracting bioactive compounds from turmeric and other plant sources.

## Figures and Tables

**Table 1:** Curcuminoid content in the extracts is numbered according to the DoE matrix.

Extract	Curcuminoid Content (μg/g)
Extract 1	7.5
Extract 2	216.6
Extract 3	91.9
Extract 4	152.4
Extract 5	183.5
Extract 6	236.9
Extract 7	163.1
Extract 8	99.6
Extract 9	107.4
Extract 10	130.3
Extract 11	164.0
Extract 12	175.8
Extract 13	199.3
Extract 14	179.2
Extract 15	181.7

**Table 2:** Curcuminoid content in the extracts was obtained using various natural deep eutectic and alcohol solvents. Columns with different superscript letters (a–i) differ significantly (*p* < 0.05).

Solvent	Hydrogen Bond Acceptor (HBA)	Hydrogen Bond Donor (HBD)	Molar Ratio (HBA:HBD)	Curcuminoid Content in the Extract (mg/g)
NADES_1	choline chloride	lactic acid	1:1	13.77 ± 0.17 ^g^
NADES_2	choline chloride	citric acid	1:1	8.22 ± 0.03 ^i^
NADES_3	choline chloride	urea	1:2	12.46 ± 0.13 ^h^
NADES_4	choline chloride	propylene glycol	1:2	23.12 ± 0.18 ^d^
NADES_5	menthol	lactic acid	1:2	30.50 ± 0.39 ^a^
NADES_6	menthol	lauric acid	2:1	17.89 ± 0.20 ^f^
NADES_7	menthol	stearic acid	8:1	19.31 ± 0.23 ^e^
NADES_8	menthol	myristic acid	8:1	25.94 ± 0.12 ^c^
80% ethanol	-	-	-	26.42 ± 0.08 ^b^
80% methanol	-	-	-	22.95 ± 0.02 ^d^

**Table 3:** Equations of logarithmic curves, correlation coefficients, and IT_50_ coefficient values for individual extract concentrations.

Invertebrate	Concentration (µL/mL)	CUR–scCO_2_–NADES_1:20		
		y = a ln(x) + b	R^2^	IT_50_ (s)
*Daphnia pulex*	0.04	y = 2.3491ln(x) − 11.017	0.9205	914
	0.10	y = 3.6549ln(x) − 18.838	0.9529	680
	0.20	y = 3.7233ln(x) − 18.203	0.9148	509
	1.00	y = 4.1438ln(x) − 21.891	0.901	658
	4.00	y = 5.6058ln(x) − 30.771	0.9113	591
	10.00	y = 5.3741ln(x) − 27.693	0.9355	439
*Chironomus aprilinus*	0.04	y = 45.974ln(x) − 518.66	0.8911	88,464 *
	0.10	y = 19.781ln(x) − 220.27	0.7598	88,274 *
	0.20	y = 14.221ln(x) − 158.17	0.6445	96,170 *
	1.00	y = 1.8697ln(x) − 16.515	0.7253	99,428 *
	4.00	y = 2.0262ln(x) − 13.344	0.609	8548
	10.00	y = 1.7811ln(x) − 7.1898	0.9066	938

* IT_50_ values calculated by extrapolation.

**Table 4:** Determined EC_50_ coefficients, logarithmic curve equation, and regression coefficient R^2^.

	CUR–scCO_2_–NADES_1:20		
	y = a ln(x) + b	R^2^	EC_50_ (µL/mL)
*Daphnia pulex*	-	-	h.t.
*Chironomus aprilinus*	y = 1.1462ln(x) + 7.6575	0.9649	0.098

h.t.—high toxicity (for all concentrations, death of 100% of organisms after 24 h).

**Table 5:** Extraction process experiment plan.

Extract	Pressure (PSI)	CO_2_ Volume (mL)	Temperature (°C)
Extract 1	2500	100	30
Extract 2	6500	175	55
Extract 3	2500	25	55
Extract 4	6500	100	30
Extract 5	2500	100	80
Extract 6	6500	100	80
Extract 7	6500	25	55
Extract 8	2500	175	55
Extract 9	4500	25	30
Extract 10	4500	25	80
Extract 11	4500	175	30
Extract 12	4500	175	80
Extract 13	4500	100	55
Extract 14	4500	100	55
Extract 15	4500	100	55

**Table 6:** Composition of natural deep eutectic solvents.

	Hydrogen Bond Acceptor	Hydrogen Bond Donor	Molar Ratio
NADES_1	choline chloride	lactic acid	1:1
NADES_2	choline chloride	citric acid	1:1
NADES_3	choline chloride	urea	1:2
NADES_4	choline chloride	propylene glycol	1:2
NADES_5	menthol	lactic acid	1:2
NADES_6	menthol	lauric acid	2:1
NADES_7	menthol	stearic acid	8:1
NADES_8	menthol	myristic acid	8:1

**Table 7:** The ratio of turmeric rhizome mass to NADES_5 volume in the cell of the supercritical carbon dioxide extractor.

Extract	Plant Material to NADES_5 Ratio (*m*/*v*)
CUR–scCO_2_–NADES_4:1	4:01
CUR–scCO_2_–NADES_2:1	2:01
CUR–scCO_2_–NADES_2:1	2:01
CUR–scCO_2_–NADES_1:2	1:02
CUR–scCO_2_–NADES_1:3	1:03
CUR–scCO_2_–NADES_1:10	1:10
CUR–scCO_2_–NADES_1:20	1:20

## Data Availability

Data are available in a publicly accessible repository.

## Supplementary Materials

The following supporting information can be downloaded at: https://www.mdpi.com/article/10.3390/ph17121596/s1, Table S1: HPLC method validation parameters.
