# Supplementary material for: Natural Deep Eutectic Solvents Combined with Supercritical Carbon Dioxide for the Extraction of Curcuminoids from Turmeric
Source: Pharmaceuticals (Basel). 2024 Nov 27;17(12):1596. doi: 10.3390/ph17121596 (PMC11676539; doi:10.3390/ph17121596)
Supplement: Supplementary file 1 [file pharmaceuticals-17-01596-s001.zip › pharmaceuticals-3342226-supplementary.pdf]

Supplementary materials

Natural deep eutectic solvents combined with supercritical carbon dioxide for the extraction of curcuminoids from turmeric

Anna Stasiłowicz-Krzemień[1,*], Julia Wójcik[1], Anna Gościniak[1], Marcin Szymański[2], Krzysztof Górecki[3], Judyta Cielecka-Piontek[1,4]

[1] Department of Pharmacognosy and Biomaterials, Poznan University of Medical Sciences, Rokietnicka 3, 60-806 Poznan, Poland; astasilowicz@ump.edu.pl (A.S-K.); agosciniak@ump.edu.pl (A.G.), jpiontek@ump.edu.pl (J.C.-P.)
[2] Center for Advanced Technologies, Adam Mickiewicz University in Poznań, Uniwersytetu Poznańskiego 9 10, 61-614, PL, marcin.szymanski@amu.edu.pl (M.Sz.)
[3] Department of Entomology and Environmental Protection, Poznan University of Life Sciences, Dąbrowskiego 159, 60-594 Poznań, Poland; krzysztof.gorecki @up.poznan.pl (K.G.)
[4] Department of Pharmacology and Phytochemistry, Institute of Natural Fibres and Medicinal Plants, Wojska Polskiego 71b, 60-630 Poznan, Poland
* Correspondence: astasilowicz@ump.edu.pl

Table S1. HPLC method validation parameters

Parameter	Curcumin	Demethoxycurcumin	Bisdemethoxycurcumin
**Calibration curve**	y =120329779.9x	y =108678736.4	y = 137539888x
**a ± Sa**	120329779.9 ±588790.54	108678736.4 ±1282251.05	137539888±854837.29
**b ± S_b**	insignificant	insignificant	insignificant
**Linearity range (μg/mL)**	10-100	2-100	5-100
**Correlation coefficient (r)**	0.999	0.999	0.999
**Limit of Detection<br><br>LOD = 3.3*SD/a (μg/mL)**	2	1	2
**Limit of quantification<br><br>LOQ = 10*SD/a (μg/mL)**	6	2	5
